# The Transcriptomic and Bioinformatic Characterizations of Iron Acquisition and Heme Utilization in *Avibacterium paragallinarum* in Response to Iron-Starvation

**DOI:** 10.3389/fmicb.2021.610196

**Published:** 2021-03-04

**Authors:** Caiyun Huo, Ximin Zeng, Fuzhou Xu, Fangbing Li, Donghai Li, Guiping Li, Zhenguo Hu, Yanxin Hu, Jun Lin, Huiling Sun

**Affiliations:** ^1^Beijing Key Laboratory for Prevention and Control of Infectious Diseases in Livestock and Poultry, Institute of Animal Husbandry and Veterinary Medicine, Beijing Academy of Agriculture and Forestry Sciences, Beijing, China; ^2^Department of Animal Science, University of Tennessee, Knoxville, TN, United States; ^3^Key Laboratory of Animal Epidemiology of Ministry of Agriculture, College of Veterinary Medicine, China Agricultural University, Beijing, China

**Keywords:** *Avibacterium paragallinarum*, iron acquisition, heme utilization, transcriptomes, differentially expressed genes

## Abstract

*Avibacterium paragallinarum* is the pathogen of infectious coryza, which is a highly contagious respiratory disease of chickens that brings a potentially serious threat to poultry husbandry. Iron is an important nutrient for bacteria and can be obtained from surroundings such as siderophores and hemophores. To date, the mechanisms of iron acquisition and heme utilization as well as detailed regulation in *A. paragallinarum* have been poorly understood. In this study, we investigated the transcriptomic profiles in detail and the changes of transcriptomes induced by iron restriction in *A. paragallinarum* using RNA-seq. Compared with the iron-sufficiency control group, many more differentially expressed genes (DEGs) and cellular functions as well as signaling pathways were verified in the iron-restriction group. Among these DEGs, the majority of genes showed decreased expression and some were found to be uniquely present in the iron-restriction group. With an in-depth study of bioinformatic analyses, we demonstrated the crucial roles of the Hut protein and DUF domain-containing proteins, which were preferentially activated in bacteria following iron restriction and contributed to the iron acquisition and heme utilization. Consequently, RT-qPCR results further verified the iron-related DEGs and were consistent with the RNA-seq data. In addition, several novel sRNAs were present in *A. paragallinarum* and had potential regulatory roles in iron homeostasis, especially in the regulation of Fic protein to ensure stable expression. This is the first report of the molecular mechanism of iron acquisition and heme utilization in *A. paragallinarum* from the perspective of transcriptomic profiles. The study will contribute to a better understanding of the transcriptomic response of *A. paragallinarum* to iron starvation and also provide novel insight into the development of new antigens for potential vaccines against infectious coryza by focusing on these iron-related genes.

## Introduction

Infectious coryza, caused by Gram-negative bacterium *Avibacterium paragallinarum*, is a severe respiratory disease of chickens that brings enormous economic losses for the poultry industry worldwide (Blackall, 1999). *A. paragallinarum* is classified into three serovars—A, B, and C—according to the Page schemes. In South Africa, it is considered as one of the most serious diseases, with C3 being the predominant serovar (Bragg et al., 1996; Boucher et al., 2014). In China, serovars A, C, and B were identified for the first time in 1987, 1995, and 2003, respectively (Zhang et al., 2003; Sun et al., 2018; Xu et al., 2019). Several factors are responsible for the pathogenicity of *A. paragallinarum*. Among them, hemagglutinin (HA) has been regarded as the most key virulence factor because of its participation in tissue adhesion (Barnard et al., 2008). Vaccination remains the most effective preventive measure against *A. paragallinarum*. However, vaccination failures always occur with the emergence of new serovars or serovar variants (Blackall, 1999; Bragg, 2005). In addition, the advent of multidrug bacterial resistance is deemed one of the most alarming situations worldwide. Therefore, it is urgent for the development of novel broad-spectrum prophylactic agents against *A. paragallinarum*. To date, the molecular mechanisms of colonization, growth, and virulence of *A. paragallinarum* in chicken upper respiratory tract are still poorly understood.

Iron is an essential micronutrient for most bacteria, which plays essential roles in bacterial colonization, growth, and virulence. Unfortunately, the concentration of free iron in the host is not enough to support the growth of bacteria (del Rio et al., 2005). In order to achieve effective iron homeostasis, bacteria have evolved sophisticated iron-acquisition systems to scavenge iron from their surroundings (siderophores, hemophores, or host-molecule-binding proteins) to ensure adequate supplies (Parrow et al., 2013). The siderophore transport system, which is capable of acquiring free inorganic iron, consists of both siderophores and TonB-dependent transferrin receptors (TBDR) on the outer membrane of bacteria (Chu et al., 2010). In *A. paragallinarum*, Abascal et al. (2009) have identified three up-expressed outer membrane proteins acting as transferrin receptor and iron transport proteins in bacteria cultured in iron-restricted medium, which are regulated by the Fur protein (Abascal et al., 2009). They speculate that these proteins are associated with the colonization of bacteria in an iron-limited host environment. Besides transferrin and lactoferrin, enterobactin is also proved to be a crucial siderophore. Studies have confirmed that the growth ability of many bacteria has been enhanced in the presence of enterobactin, including not only enteric bacteria such as *Escherichia coli* and *Campylobacter jejuni*, but also respiratory bacteria such as *Hemeophilus parainfluenzae* and *Bordetella pertussis* (Williams et al., 1990; Thulasiraman et al., 1998; Anderson and Armstrong, 2004; Horiyama and Nishino, 2014). In terms of ferric enterobactin receptor, we have previously demonstrated that CfrA and CfrB contribute to iron acquisition in *Campylobacter* (Zeng et al., 2009; Xu et al., 2010). Until now, the mechanisms of iron acquisition by siderophores and the detailed regulation in *A. paragallinarum* are still on the preliminary stage and remain to be further explored.

Remarkably, the availability of free iron is strongly limited in vertebrate hosts, causing insufficient iron acquisition by siderophores, especially for pathogenic bacteria (Sekine et al., 2016). Thus, the heme utilization system has been well developed in bacteria to better obtain iron. Over the past decades, the utilization of heme is validated to be a common mechanism employed by pathogens. For example, heme is preferred as an iron source over ferrous iron for some Gram-positive bacteria such as *Staphylococcus aureus* and *Streptococcus pyogenes* (Rouault, 2004; Skaar et al., 2004; Zhu et al., 2008). For Gram-negative pathogens, proteins encoded within the heme transport operon have been studied such as *Vibrio anguillarum* (Sekine et al., 2016). To acquire heme from hemoproteins, bacteria have developed sophisticated heme acquisition machineries. Gram-negative pathogens can also utilize an outer TonB-dependent membrane receptor protein to transport heme into the periplasm, and then periplasmic heme-binding proteins transport heme to the cytoplasm (Contreras et al., 2014; Richard et al., 2019). In the cytoplasm, heme can be degraded by heme-degrading enzymes, which remove iron from the heme (Sekine et al., 2016). Nevertheless, the research on heme uptake and utilization by *A. paragallinarum* is rare.

Small regulatory RNAs (sRNAs) act as post-transcriptional regulators controlling bacterial adaptation to environmental changes (Koeppen et al., 2016; Gerrick et al., 2018). Their regulatory functions have become the center of attention in a variety of bacteria, such as *E. coli*, *Salmonella*, and *Pseudomonas aeruginosa* (Koeppen et al., 2016; Chareyre and Mandin, 2018; El Mouali et al., 2018). sRNAs play crucial roles in regulating outer membrane protein expression, iron ion balance, community induction, and bacterial pathogenicity. The regulatory mechanism involves binding to target bacterial mRNAs or directly interact with target proteins (Repoila and Darfeuille, 2009). For instance, sRNAs RyhB of enteric bacteria and PrrF of *P. aeruginosa* are highly upregulated during iron starvation, which are able to suppress mRNAs encoding iron-containing proteins (Masse et al., 2005; Reinhart et al., 2015). Recently, MrsI is identified to be important for iron-sparing response in *Mycobacterium tuberculosis* (Gerrick et al., 2018). To date, sRNA studies in *A. paragallinarum* have been limited, let alone the interaction between any sRNA and its targets.

The identification and quantification of differentially expressed genes (DEGs) can be conducted at a transcriptomic level by using RNA sequencing (RNA-seq) technology. This transcriptomic approach has identified 13 upregulated genes encoding for proteins exposed to iron-restriction stress at the bacterial surface of *Hemeophilus* (*Glässerella*) *parasuis* (Alvarez-Estrada et al., 2018). In addition, RNA-seq can also be used to characterize sRNAs, including their secondary structure and predicted mRNA targets. Koeppen et al. (2016) have characterized differentially packaged sRNAs in outer membrane vesicle secreted by *P. aeruginosa*. Nevertheless, a comprehensive analysis of the transcriptomic changes in *A. paragallinarum* to iron-restriction stress has yet to be performed.

To get a deeper understanding of the biological pathways and main transcripts involved in iron-restriction stress as well as exploring the specific molecular mechanism behind iron acquisition and heme utilization in *A. paragallinarum*, we used RNA-seq for gaining information about gene and sRNA abundance in *A. paragallinarum* under iron-restriction condition. To the best of our knowledge, this is the first report regarding the transcriptomic and bioinformatic characterizations of iron acquisition and heme utilization in *A. paragallinarum* in response to iron starvation.

## Materials and Methods

### Bacterial Strain and Growth Conditions

The strain 3005 of the *A. paragallinarum* serogroup C was used in this study, which was isolated from China in 2018. As outlined in Supplementary Figure 1, tryptic soya broth (TSB) and tryptic soya broth agar (TSA) that were added with 10% chicken serum and 0.0025% nicotinamide adenine dinucleotide (NAD) were used for the propagation and maintenance of the strain. Then, *A. paragallinarum* was grown under *in vitro* culture conditions with iron restriction (R) or without iron restriction (C, as a control). For the iron-restriction condition, *A. paragallinarum* cells were inoculated at 1:100 in 200 ml minimal essential medium alpha (MEMα) (Invitrogen) with 0.0025% (w/v) NAD (Palyada et al., 2004). For the control condition, an extra 40 μM FeSO_4_ was added in the cultures based on the related reference (Palyada et al., 2004). The cells were grown in a shaking incubator at 37°C until reaching an optical density (OD) of 0.6-1.0 at 600 nm (exponential phase of growth), and samples were harvested by centrifugation, followed immediately by being resuspended in RNA later (Ambion, Carlsbad, CA, United States). Samples were then stored at −80°C. Three replicates were made under each condition.

### RNA Extraction and Sample Preparation

Total RNA for RNA sequencing was extracted using TRIzol^®^ reagent (Invitrogen) according to the manufacturer’s instructions, and the contaminating DNA was removed by treatment with DNase I (TaKaRa) and confirmed by negative PCR amplification results. Finally, RNA quality was determined by 2100 Bioanalyser (Agilent) and quantified using the ND-2000 (NanoDrop Technologies).

### Library Preparation and Illumina Sequencing

Two micrograms of total RNA from each sample was used for RNA-seq transcriptome library preparation. After ribosomal RNA (rRNA) was depleted, mRNA was purified and fragmented. Subsequently, first- and second-strand cDNAs were synthesized and ligated with adapters. cDNA library preparation was performed sequentially with the TruSeq RNA Sample Prep Kit from Illumina (San Diego, CA, United States) following the manufacturer’s protocol. After quantified by TBS380 (Picogreen), paired-end RNA-seq sequencing library was sequenced with the Illumina HiSeq × 10 (2 × 150 bp read length). The processing of original images to sequences, base-calling, and quality value calculations were performed using the Illumina GA Pipeline (version 1.6), in which 150 bp paired-end reads were obtained. A Perl program was written to select clean reads by removing low-quality sequences, reads with more than 5% of N bases (unknown bases), and reads containing adaptor sequences.

### Mapping and Alignment of Sequence Reads

After the low-quality reads were trimmed and the adapters were removed, the clean reads were mapped and aligned onto the whole reference genome (*A. paragallinarum* strain AVPG2015, GenBank accession nr NC_011852.1) using Bowtie 2.0.

### Differential Gene Analysis of RNA-Seq

The raw data were processed for the gene data analysis using the normalized transcripts per million mapped reads (TPM) values using RSEM, and differential gene expression was obtained by DESeq2. Based on the negative binomial distribution model, heterogenetic analysis among samples was obtained by controlling the fold change (FC) and *p* value statistical methods. The screening conditions of this study were | log2FC| ≥ 1 and *p* < 0.05. An analysis of variance (ANOVA), using the Benjamini–Hochberg multiple testing correction, was performed to identify genes significantly differentially expressed (*p* < 0.05). Significantly differential expressed genes with fold change ≥ 2 were subjected to further Gene Ontology (GO) and pathway analysis.

### Bioinformatics and Statistical Analyses

Differentially expressed genes were classified for the categories using annotations of GO and Kyoto Encyclopedia of Genes and Genomes (KEGG) pathways with the DAVID online software^1^. The threshold of significantly enriched genes was determined according to the corrected *p* (Benjamini) (*p* ≤ 0.05). The default parameters (hypergeometric algorithm, Benjamini–Hochberg multiple testing correction) were performed to take overrepresentation analyses. GO terms (biological process, cellular component, and molecular function) and KEGG pathways terms with *p*-Values of ≤0.05 were considered to be significantly enriched with DEGs. The heatmaps of DEGs were achieved with the pheatmap packages from R.

### sRNA Analysis

Small regulatory RNAs prediction was obtained by the current mainstream prokaryotic analysis software Rockhopper^2^, which was mainly based on the base sequencing coverage information. Besides, the RNAfold program in the Vienna RNA packages was used to predicate the secondary structure of sRNA based on the minimum free energy.

### Data Deposition

The raw sequencing files from this study were deposited into the NCBI SRA repository and the SRA accession number was SRR13291129.

### Validation of DEGs by Quantitative Real-Time PCR

Differentially expressed genes identified by the above-described method were validated using quantitative real-time PCR (qPCR). The gyrA was used as a reference control (Wen et al., 2016). The information on the primers is listed in Supplementary Table 1. The qPCR programs for VY92_RS06600, VY92_RS09125, VY92_RS09790, VY92_RS03730, and VY92_RS03735 were as follows: 1 cycle of 95°C for 3 min; 35 cycles of 95°C for 30 s, 60°C for 30 s, and 72°C for 40 s. The qPCR programs for VY92_RS01655, VY92_RS00335, VY92_RS05725, and gyrA were as follows: 1 cycle of 95°C for 3 min; 35 cycles of 95°C for 30 s, 56°C for 30 s, and 72°C for 40 s. Gene expression levels were calculated as previously described (Huo et al., 2020). Data analysis was conducted by using two-way analysis of variance with GraphPad Prism (ver. 5.0). *p* < 0.05 represents statistical significance. The results were shown as the means ± standard deviations of three independent experiments.

## Results

### Quality Control of Sequencing Data and Mapping

As outlined in Supplementary Figure 1, the cells were grown and harvested at 5 h for RNA sequencing based on the measurement of OD values *via* bacteria growth assay (Supplementary Table 2). Then, the quality control of sequencing data was taken. As shown in Table 1, 13.70 to 16.68 M raw reads were generated for each sample, which the RawQ20 and Raw30 ratios were all above 90%. High-quality reads were obtained and used for further analyses after quality assessment using the FastQC tool and data preprocessing. After the quality control, clean reads with numbers averaging between 13.00 and 15.87 M were obtained per sample, in which the CleanQ20 and CleanQ30 ratios reached more than 95%. Besides, we also assessed the nucleotide distribution of both raw and clean data and found that sequences were all roughly distributed at about 150 bp (Supplementary Figure 2).

**TABLE 1:** Statistics of RNA-seq data.

Sample	R_1	R_2	R_3	C_1	C_2	C_3
Raw reads	15799798	15335136	14391550	14351858	13699662	16678476
Raw Bases (bp)	2385769498	2315605536	2173124050	2167130558	2068648962	2518449876
Raw Error Rate (%)	0.0123	0.0125	0.0125	0.0155	0.0127	0.0124
Raw Q20 (%)	97.4	97.17	97.09	94.37	96.95	97.24
Raw Q30 (%)	94.63	94.4	94.31	90.83	94.1	94.47
Clean Reads	15056962	14600584	13673204	13084052	12990990	15872350
Clean Bases (bp)	1882408242	1778991741	1653375486	1445577855	1580075894	1958144714
Clean Error Rate (%)	0.0106	0.0106	0.0106	0.0106	0.0106	0.0106
Clean Q20(%)	99.05	99.09	99.09	99.08	99.09	99.08
Clean Q30(%)	97.09	97.18	97.19	97.16	97.16	97.14

*R_1, R_2, and R_3 represent the three independent replicates in iron-restriction group, C_1, C_2, and C_3 represent the three independent replicates in control group. Q20 = bases of Q ≥ 20/all bases of sequencing. Q30 = bases of Q ≥ 30/all bases of sequencing.*

**FIGURE 1:**
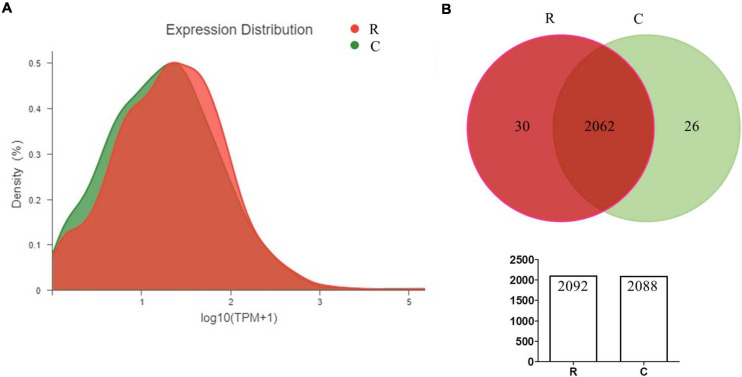
Quantitative statistics of gene expression **(A,B)**. The density diagram and Venn diagram showing the distribution of gene expression and the overlap of genes in *A. paragallinarum* under conditions with iron restriction or without iron restriction, respectively.

In order to quantify the gene expression in samples, sequence mapping and alignment analysis of high-quality clean reads was taken at first by comparing with the reference genome (*A. paragallinarum* strain AVPG2015, GenBank accession nr NC_011852.1). As shown in Table 2, more than 80% of reads from all experimental groups could be aligned to the reference genome, and unique mapped reads ratio ranged from 46.32 to 76.1%. Besides, 81.69 to 87.63% of these clean reads were distributed in coding regions. The distribution and numbers of gene expression are shown in Figure 1A. In the density diagram of gene expression, high- and low-abundance genes could be shown and formed an obvious peak in each group. Furthermore, the TPM of genes was shown to be similar between these two groups, indicating the stability of the expression levels. Thus, the sequencing data conformed to the requirements of subsequently bioinformatics analysis.

**TABLE 2:** Statistics of mapping.

Sample	R_1	R_2	R_3	C_1	C_2	C_3
Total Reads	15056962	14600584	13673204	13084052	12990990	15872350
Genome Mapped Reads	12832784	12362254	11337669	11422710	11539198	14028953
Genome Mapped Ratio(%)	85.23	84.67	82.92	87.3	88.82	88.39
Unmapped Reads	2224178	2238330	2335535	1661342	1451792	1843397
Unmapped Reads Ratio(%)	14.77	15.33	17.08	12.7	11.18	11.61
Uniq Mapped Reads	11457943	10097739	9241254	6059944	9453081	11940812
Uniq Mapped Reads Ratio(%)	76.1	69.16	67.59	46.32	72.77	75.23
CDS Mapped Reads	12555029	12177510	11169076	11311302	11383783	13805584
CDS Mapped Ratio(%)	83.38	83.4	81.69	86.45	87.63	86.98

*R_1, R_2, and R_3 represent the three independent replicates in iron-restriction group, C_1, C_2, and C_3 represent the three independent replicates in control group.*

### Identification and Quantification of Differentially Expressed Genes

According to the quantification of gene expressions, we analyzed 2,566 transcripts, including 2,445 genes encoding proteins and 121 genes encoding sRNAs (Supplementary Data Sheet 1). By taking the Venn analysis, we found that there were 2,062 common genes in these two groups (Figure 1B). In addition, 30 genes were uniquely expressed in *A. paragallinarum* under iron-restriction condition and 26 genes were uniquely expressed in *A. paragallinarum* under the control condition, respectively.

**FIGURE 2:**
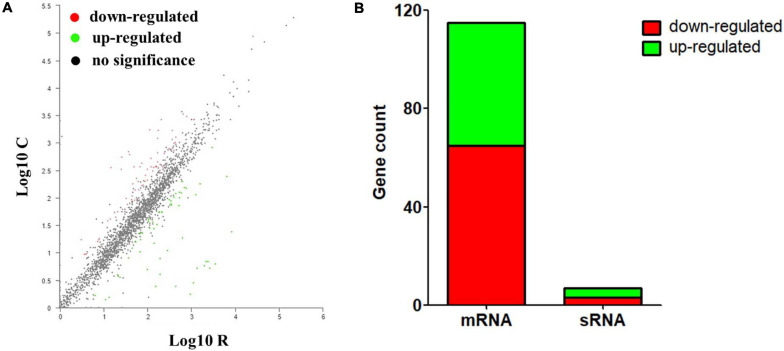
Identification and quantification comparison of differentially abundant genes in *A. paragallinarum* under iron-restriction condition compared with the control condition. **(A)** Scatter plots of gene abundance ratio in iron-restriction versus control conditions. **(B)** Bar chart showing upregulated differentially expressed genes (DEGs) (green) and downregulated DEGs (red) in *A. paragallinarum* under iron-restriction condition compared with the control condition (iron restriction vs control).

Based on the criteria of | log2FC| ≥ 1 and *p*-adjust < 0.05, the numbers of genes that up- and downregulated their abundance were summarized. As shown in Figure 2 and Supplementary Data Sheet 1, 122 DEGs were identified significantly between the iron-restriction group and the control group. Compared with the iron-sufficiency control group, iron restriction could significantly downregulate 68 genes, including 65 genes encoding for proteins and 3 genes encoding for sRNAs, which include autotransporter outer membrane beta-barrel domain-containing protein and other proteins. Meanwhile, 54 genes were significantly upregulated in the iron-restriction condition than in the control, which consisted of 50 genes encoding for proteins and 4 genes encoding for sRNAs. Among these 54 differentially upregulated genes, 19 genes could directly evolve into sophisticated iron-acquisition systems, such as iron utilization receptors, ABC transporters, and TonB energy systems (Table 3). Additionally, several iron-regulated operons were identified for the gene clustering analysis, suggesting that a number of differentially up-expressed genes might be cotranscribed. There were several operons involved in ABC transporters: one that contained hemoglobin/transferrin/lactoferrin receptor (VY92_RS00325) and ABC transporter ATP-binding protein (VY92_RS00330 and VY92_RS00335), one that contained Fe(3+)-hydroxamate ABC transporter permease FhuB (VY92_RS05675) and iron-hydroxamate transporter ATP-binding subunit (VY92_RS05685), and another one that contained ABC transporter ATP-binding protein (VY92_RS05745) and ABC transporter permease (VY92_RS05750). An operon was shown to be involved in heme utilization systems that contained heme utilization protein HutZ (VY92_RS03730), heme utilization cytosolic carrier protein HutX (VY92_RS03735), and TonB-dependent receptor (VY92_RS03740). An operon was shown to be involved in iron transporter and iron permease that contained iron transporter (VY92_RS05765), iron permease (VY92_RS05770), and iron-sulfur cluster carrier protein ApbC (VY92_RS05775). There was also an operon that was involved in TonB energy systems, containing energy transducer TonB (VY92_RS11145) and TonB system transport proteins ExbD and ExbB (VY92_RS11150 and VY92_RS11155). Interestingly, 98 genes encoding for numerous domains of unknown function (DUF) domain-containing proteins were discovered in *A. paragallinarum*. Among these genes, there were 57 (58%) upregulated and 17 (17%) downregulated DUF genes in the iron-restriction group. Furthermore, significant differences of gene expressions were shown in three upregulated DUF genes, which encoded DUF454, DUF4198, and DUF2318 domain-containing proteins, respectively. There was also one DUF gene that encodes DUF1345 domain-containing protein that was significantly downregulated in the iron-restriction group than in the control group.

**TABLE 3:** Summary of 19 differentially up-regulated genes that could directly evolve into sophisticated iron-acquisition systems in *Av. paragallinarum* under iron-restriction condition compared to control condition.

Gene_id	Gene description	Fold change	*P*-value
VY92_RS00325	TonB-dependent hemoglobin/transferrin/lactoferrin family receptor	9.23	2.3574E-61
VY92_RS00330	ABC transporter ATP-binding protein	8.51	6.7815E-100
VY92_RS00335	ABC transporter ATP-binding protein	8.28	1.1651E-108
VY92_RS03730	heme utilization protein HutZ	8.47	8.557E-161
VY92_RS03735	heme utilization cytosolic carrier protein HutX	9.25	6.7245E-117
VY92_RS03740	TonB-dependent receptor	9.38	1.07492E-67
VY92_RS04845	TonB-dependent receptor	8.24	3.53924E-83
VY92_RS05675	Fe(3 +)-hydroxamate ABC transporter permease FhuB	2.94	0.001252357
VY92_RS05685	iron-hydroxamate transporter ATP-binding subunit	2.72	0.000847211
VY92_RS05745	ABC transporter ATP-binding protein	1.95	0.00208536
VY92_RS05750	ABC transporter permease	1.85	0.000148102
VY92_RS05765	iron transporter	4.72	1.96695E-21
VY92_RS05770	iron permease	3.33	2.83576E-21
VY92_RS05775	iron-sulfur cluster carrier protein ApbC	1.41	0.001180495
VY92_RS07080	TonB-dependent receptor	6.75	4.16422E-15
VY92_RS08670	ferric iron uptake transcriptional regulator	2.23	6.50E-10
VY92_RS11145	energy transducer TonB	4.64	8.04801E-16
VY92_RS11150	TonB system transport protein ExbD	5.96	1.33945E-24
VY92_RS11155	TonB-system energizer ExbB	5.16	5.65457E-12

In the case of 115 significantly DEGs encoding proteins, the hierarchical cluster analysis of these DEGs was carried out for understanding the relationships between genes across all 6 samples (Supplementary Figure 3). Above all, it suggests that iron restriction could result in significant changes of gene expressions in *A. paragallinarum* and seems to be likely to suppress expressions of the majority of genes in bacteria.

### Functional Annotations and Bioinformatic Characterization of DEGs in *A. paragallinarum* Under Iron-Restriction Condition Using GO Enrichment Analysis

**FIGURE 3:**
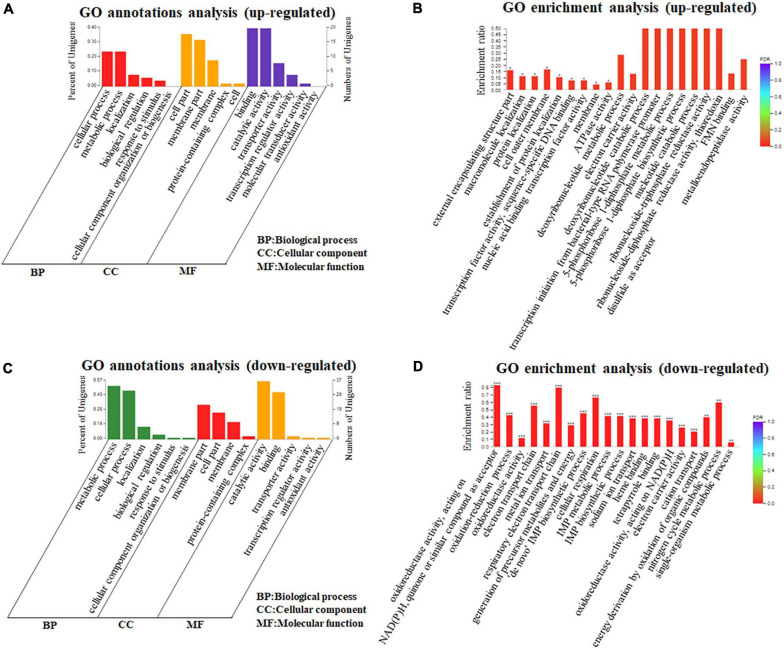
Gene Ontology (GO) annotations and enrichment analysis of DEGs in *A. paragallinarum* under iron-restriction condition compared with the control condition. **(A,B)** GO annotations and enrichment analysis of upregulated genes were analyzed, respectively. **(C,D)** GO annotations and enrichment analysis of downregulated genes were analyzed, respectively (*P < 0.05; **P < 0.01; ***P < 0.001).

Based on the identification and quantification of DEGs, we further performed the functional annotations and bioinformatic characterization of DEGs in *A. paragallinarum* under iron restriction using GO enrichment analysis through the DAVID online analysis system. Firstly, we took the GO annotations analysis of these DEGs. As shown in Figure 3A and Supplementary Data Sheet 2, upregulated genes could be clustered into three categories, including six kinds of biological processes, five kinds of cellular components, and six kinds of molecular functions. Then, the GO enrichment analysis of these upregulated genes was carried out and the top 20 terms are summarized in Figure 3B and Supplementary Data Sheet 3. Twenty-two upregulated genes enriched in the 13 GO biological process terms, 22 upregulated genes enriched in the 5 GO cellular component terms, and 45 upregulated genes actively participated in the 27 GO molecular function terms were present in *A. paragallinarum* under the iron-restriction condition, respectively. Among them, three biological process terms that were involved in the response to macromolecule localization, protein localization, and establishment of protein localization were significantly enriched. Another three cellular component terms for protein binding were significantly enriched, which were mainly involved in the cell outer membrane, external encapsulating structure part, and membrane. Besides, biological process items for the transcription factor activity and sequence-specific DNA binding, nucleic acid binding transcription factor activity, and ATPase activity were significantly enriched.

In addition, the functional annotations and bioinformatic characterization of downregulated genes in *A. paragallinarum* under the iron-restriction condition were taken by GO enrichment analysis. As shown in Figure 3C and Supplementary Data Sheet 2, the downregulated genes could also be classified into three categories, which consisted of six kinds of biological processes, four kinds of cellular components, and five kinds of molecular functions. The GO enrichment analysis of these downregulated genes was also conducted and the top 20 terms are summarized in Figure 3D and Supplementary Data Sheet 4. The data showed that there were 391 downregulated genes enriched in the 75 GO biological process terms, 54 downregulated genes enriched in the 8 GO cellular component terms, and 185 downregulated genes actively participated in the 50 GO molecular function terms in *A. paragallinarum* under the iron-restriction condition, respectively. Additionally, 14 biological process items and 6 molecular function items were significantly enriched, which mainly participated in the oxidation–reduction process; electron transport chain; respiratory electron transport chain; generation of precursor metabolites and energy; “*de novo*” inosine-5’-monophosphate (IMP) biosynthetic process; cellular respiration; IMP metabolic process; IMP biosynthetic process; sodium ion transport; cation transport; energy derivation by oxidation of organic compounds; nitrogen cycle metabolic process; single-organism metabolic process; metal ion homeostasis; oxidoreductase activity; acting on NAD(P)H, quinone, or similar compound as acceptor; heme binding; tetrapyrrole binding; acting on NAD(P)H; and electron carrier activity. However, no cellular component item was significantly enriched for these downregulated genes. Taken together, the identification of a larger number of DEGs between the iron-restriction group and the control group using DEGs GO enrichment analysis suggests that iron restriction can result in changes of a variety of biological processes and cellular component terms as well as molecular functions that are related to DEGs in *A. paragallinarum*.

### Functional Annotations and Bioinformatic Characterization of DEGs in *A. paragallinarum* Under Iron-Restriction Condition Using KEGG Pathway Enrichment Analysis

**FIGURE 4:**
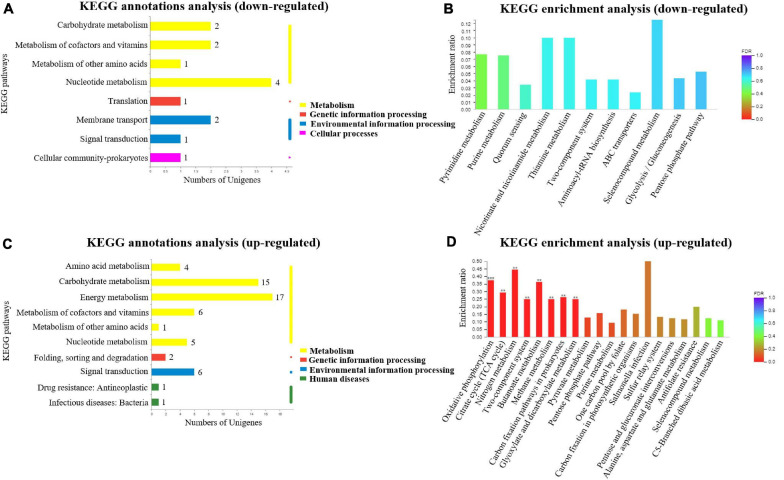
Kyoto Encyclopedia of Genes and Genomes (KEGG) annotations and enrichment analysis of DEGs in *A. paragallinarum* under iron-restriction condition compared with the control condition. **(A,B)** KEGG annotations and enrichment analysis of upregulated genes were performed, respectively. **(C,D)** KEGG annotations and enrichment analysis of downregulated genes were performed, respectively (**P < 0.01; ***P < 0.001).

In order to further detect the related pathways in which these DEGs in *A. paragallinarum* under the iron-restriction condition were involved in, the KEGG pathway analysis was performed. As shown in Figure 4A, upregulated genes could be classified into four KEGG categories, including four kinds of metabolism, one kind of genetic information processing, two kinds of environmental information processing, and one kind of cellular process. Then, KEGG enrichment analysis of these upregulated genes was carried out. As shown in Figure 4B and Supplementary Data Sheet 5, 17 upregulated genes were enriched in the 11 KEGG pathway items such as ABC transporters, pyrimidine metabolism, purine metabolism, quorum sensing, nicotinate and nicotinamide metabolism, and so on. However, no KEGG pathway item was significantly enriched.

By taking KEGG pathway analysis of downregulated genes in *A. paragallinarum* under iron-restriction condition, we found that the downregulated genes could also be classified into four KEGG categories, including six kinds of metabolism, one kind of genetic information processing, one kind of environmental information processing, and two kinds of human diseases (Figure 4C). Furthermore, KEGG enrichment analysis showed that 75 downregulated genes were enriched in the 30 KEGG pathway items. Eight of them exhibited dramatic enrichment that were related to oxidative phosphorylation, citrate cycle (TCA cycle), nitrogen metabolism, two-component system, butanoate metabolism, methane metabolism, carbon fixation pathways in prokaryotes, and glyoxylate and dicarboxylate metabolism (Figure 4D and Supplementary Data Sheet 6). Above all, the results of KEGG enrichment analysis demonstrate that iron restriction can result in changes of a series of biological metabolic pathways and signal transduction pathways that are associated with DEGs in *A. paragallinarum*.

### Validation of DEGs in *A. paragallinarum* Under Iron-Restriction Condition Through qPCR Analysis

**FIGURE 5:**
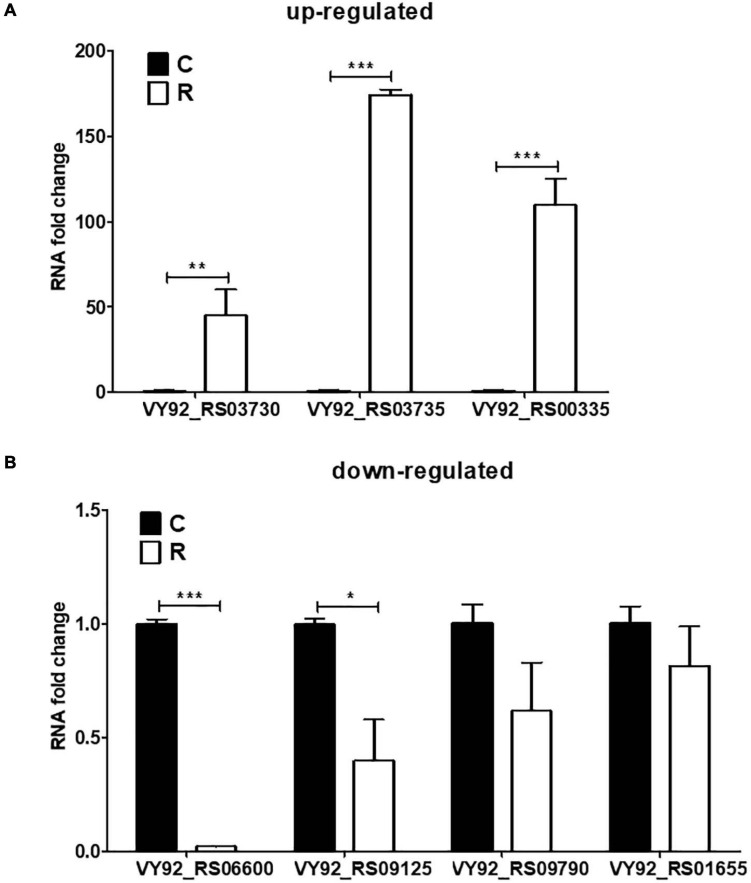
Validation of DEGs in *A. paragallinarum* under iron-restriction condition compared with the control condition by qPCR. **(A,B)** The mRNA levels of three upregulated genes and four downregulated genes in *A. paragallinarum* under iron-restriction condition compared with the control condition were determined by real-time PCR (*N* = 3), respectively. *p < 0.05, **p < 0.01, ***p < 0.001.

Based on the transcriptomic results, some crucial genes involved in the iron homeostasis showed significant difference between the iron-restriction group and the control group. In order to further validate the results, qPCR was carried out to detect the expressions of up- and downregulated genes in *A. paragallinarum* under iron-restriction condition. Here, three upregulated genes (VY92_RS03730, VY92_RS03735, and VY92_RS00335) and four downregulated genes (VY92_RS06600, VY92_RS09125, VY92_RS09790, and VY92_RS01655) were selected as representative DEGs for qPCR. Among them, VY92_RS03730, VY92_RS03735, and VY92_RS00335 were annotated as the heme utilization protein HutZ, heme utilization cystosolic carrier protein HutX, and ABC transporter ATP-binding protein, respectively. VY92_RS06600, VY92_RS09125, VY92_RS09790, and VY92_RS01655 were annotated as the H-type ferritin, cytochrome c nitrite reductase pentaheme subunit, succinate dehydrogenase/fumarate reductase iron–sulfur subunit, and autotransporter outer membrane beta-barrel domain-containing protein, respectively. As shown in Figure 5A, the iron-restriction group showed significantly increased mRNA levels of all three upregulated genes than the control group. In addition, the expressions of four downregulated genes were lower in the iron-restriction group than in the control group, and a significant difference could be seen in VY92_RS06600 and VY92_RS09125 (Figure 5B). Taken together, the results are in accordance with the above RNA-seq and suggest the distinct gene expressions of these iron-related DEGs in *A. paragallinarum* under iron restriction.

### Identification and Bioinformatic Characterization of sRNAs in *A. paragallinarum* Under Iron-Restriction Condition

**FIGURE 6:**
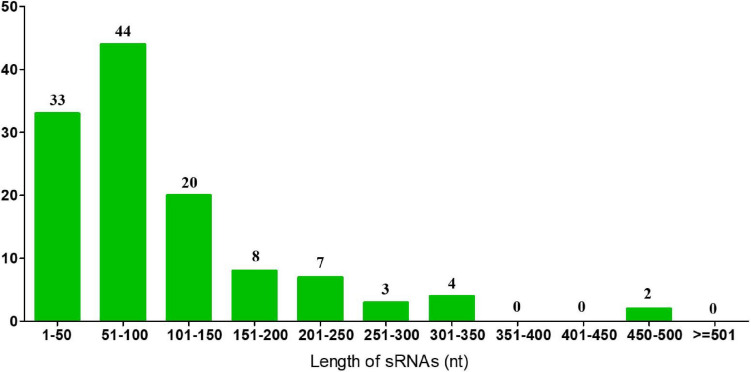
Identification of sRNAs in *A. paragallinarum*. The length of all sRNAs in *A. paragallinarum* was bioinformatically determined.

**FIGURE 7:**
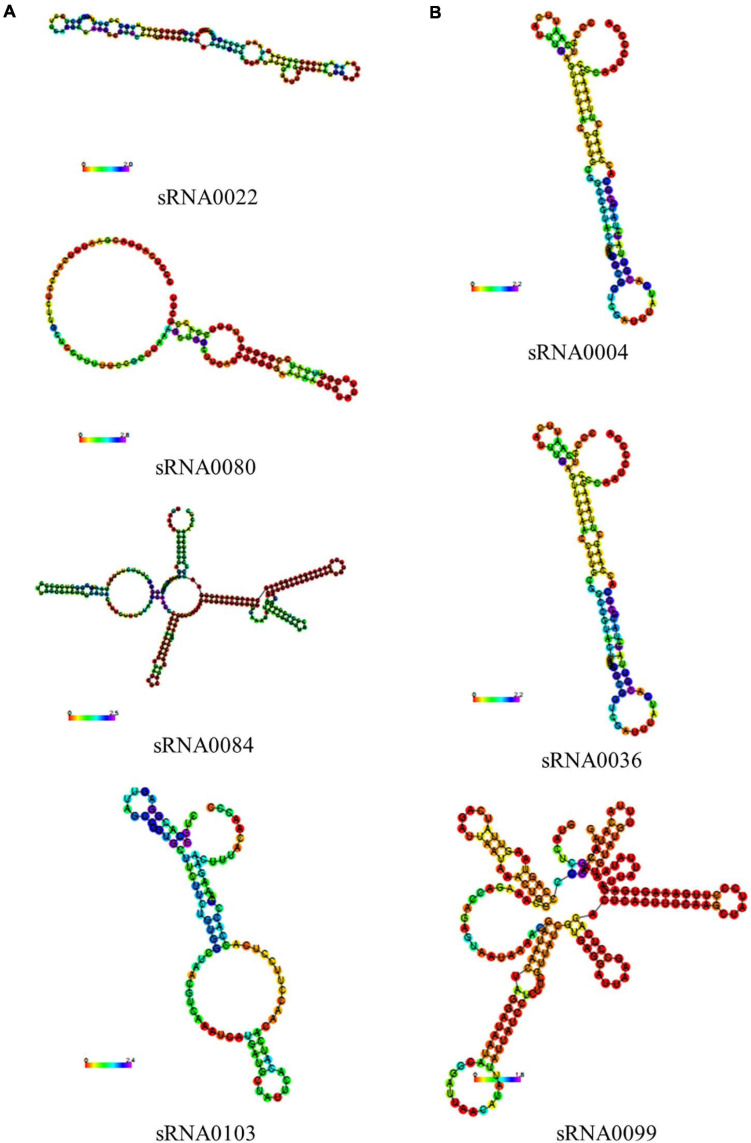
Prediction of secondary structures of several crucial sRNAs in *A. paragallinarum* under the iron-restriction condition. **(A,B)** The secondary structures of four upregulated genes and three downregulated sRNAs in *A. paragallinarum* under the iron-restriction condition compared with the control condition were predicted, respectively.

In previous results of the identification and quantification of genes in this study, 121 genes encoding sRNAs were verified. We further performed bioinformatic characterization of these sRNAs in *A. paragallinarum* under the iron-restriction condition. Firstly, we summarized the length of these sRNAs. As shown in Figure 6 and Supplementary Data Sheet 7, the length of each sRNA was smaller than 500 nt, and most of them had the length of 101 to 150 nt. Subsequently, we determined if these sRNAs were likely to form stable secondary structures. Here, we selected four upregulated sRNAs (sRNA0022, sRNA0080, sRNA0084, and sRNA0103) and three downregulated sRNAs (sRNA0004, sRNA0036, and sRNA0099) in *A. paragallinarum* under the iron-restriction condition. As shown in Figure 7, these sRNAs could form desirable stem-loop secondary structures.

It is widely known that sRNA executes its regulatory function by pairing and binding to its target genes. We also identified potential interactions between these sRNAs and target mRNAs. As shown in Supplementary Data Sheet 8, a single sRNA could target various mRNAs, and the same target mRNA may also be regulated by different sRNAs. Among the four upregulated sRNAs, we found that they could target the genes mainly encoding the proteins such as DUF3277 domain-containing protein, Fic family protein, DUF1043 domain-containing protein, DUF1828 domain-containing protein, ABC transporter permease, ABC transporter ATP-binding protein, spermidine/putrescine ABC transporter permease, heme-binding protein, DNA helicase RecQ, DNA-binding protein, DNA repair protein RecO, cAMP-activated global transcriptional regulator CRP, transcriptional regulator MraZ, succinate dehydrogenase/fumarate reductase iron–sulfur subunit, autotransporter domain-containing protein, MurR/RpiR family transcriptional regulator, met regulon transcriptional regulator MetJ, and so on. In terms of three downregulated sRNAs, sRNA0004 and sRNA0036 tended to target the genes which coded for the proteins such as DUF1367 domain-containing protein, Fic family protein, two-component system sensor histidine kinase QseC, and so on. Interestingly, we could see that genes encoding the same protein such as DUF1367 domain-containing protein could be targeted by both upregulated sRNAs and downregulated sRNAs. Unfortunately, no target gene was detected for sRNA0099 in this study. Considering that the gene encoding for the Fic protein showed no significance of transcriptomic changes of expression between the iron-restriction group and the control group as well as being targeted by the both up- and downregulated sRNAs, we also used qPCR to further detect the mRNA levels of the related gene. As shown in Supplementary Figure 4, the results were also consistent with the above RNA-seq and showed no difference of mRNA levels of VY92_RS05725 encoding for the Fic protein between the iron-restriction group and the control group, which suggested the stable expressions of Fic protein in *A. paragallinarum* under iron restriction.

Furthermore, the potential interaction between sRNAs and qPCR-validated DEGs was examined. Among those eight DEGs, VY92_RS09790 and VY92_RS00335 were shown to be targeted by various sRNAs, especially VY92_RS09790 (Table 4). VY92_RS09790, one of the crucial downregulated genes in *A. paragallinarum* under the iron-restriction condition, could be targeted by 13 sRNAs, including upregulated sRNA0022 and sRNA0103. Notably, upregulated VY92_RS00335 could also be targeted by sRNA0022, indicating the potential regulatory role of sRNA0022 in *A. paragallinarum* under the iron-restriction condition. Taken together, these results validate that sRNAs have a potential role in the regulation of iron homeostasis in *A. paragallinarum*.

**TABLE 4:** The regulatory sRNAs targeting the iron-related genes in *Av. paragallinarum*.

Target Gene ID	Description	sRNA ID
VY92_RS09790	succinate dehydrogenase/fumarate reductase iron-sulfur subunit	sRNA0003, sRNA0021, sRNA0022, sRNA0025, sRNA0037,
		sRNA0051, sRNA0054, sRNA0057, sRNA0087, sRNA0095,
		sRNA0098, sRNA0101, sRNA0103
VY92_RS00335	ABC transporter ATP-binding protein	sRNA0002, sRNA0007, sRNA0022, sRNA0033, sRNA0086,
		sRNA0104, sRNA0109

## Discussion

Recently, transcriptomic and bioinformatic analyses, as a new frontier developed in life science, have been extensively used to study dynamic alterations in global transcriptomic profiles and explore the transcriptome response of bacteria to stimuli including nutritional stress (Ying et al., 2015). Therefore, we took RNA-seq to better understand gene expression profiles in *A. paragallinarum* under the iron-restriction condition. Here, we focused on the large quantities of bacterial genes regulated by iron restriction as well as possible functional activities. These analyses will be helpful for the in-depth realization of the molecular mechanisms involved in response to nutritional iron availability in *A. paragallinarum* to control cellular iron homeostasis when faced with iron starvation.

Bacteria are generally adaptable to changing circumstances such as various nutrient deficiencies or stresses. This adaption results in many physiological changes such as expression changes of global genes and induction of specific genes (Siegele and Kolter, 1992). Here, we also evaluated and compared the DEGs in *A. paragallinarum* under conditions with iron restriction and iron sufficiency as well as the related cellular functions and signaling pathways. Between these two conditions, we have revealed a number of distinct characteristics of global gene expression in bacteria. In comparison with iron sufficiency, iron restriction could lead to both upregulation and downregulation of genes, and it seems that the majority of genes showed decreased expression. Additionally, some genes were found to be uniquely present in the iron-restriction group. As previously reported, perhaps reduction of global gene expression and induction of unique genes are also helpful for *A. paragallinarum* to enter stationary phase by ceasing growth and retaining survivability for a long time (Miksch and Dobrowolski, 1995). Notably, several upregulated genes are demonstrated to be able to facilitate *A. paragallinarum* to effectively obtain iron for their growth (Abascal et al., 2009). In our study, when bacteria are in an iron-restriction environment, these upregulated gene products that we found can also be helpful for the release of iron from iron-binding proteins and compounds, promoting the acquisition and use of more iron sources by bacteria. The outer membrane receptors are generally induced by iron restriction and thought to enhance the rate of ferric-siderophore uptake, allowing bacteria to more effectively scavenge ferric-siderophores from their surroundings. Additionally, transport of ferric-siderophores through outer membrane receptors requires energy, and this energy is provided by the TonB energy system (Postle, 1993; Larsen et al., 1994; Higgs et al., 1998). Therefore, the increased expression of some proteins that are related to the TonB energy system in bacteria under the iron-restricted condition can facilitate the entry of ferric-siderophores across the outer membrane into the periplasm. TonB ExbB-ExbD proteins are found in many Gram-negative bacteria, mostly known to be found in the *E. coli* system (Braun et al., 1998; Palyada et al., 2004). In our study, the TonB system transport proteins ExbD and ExbB were found to be either iron stimulated and cotranscribed, and it is likely that the formation of the protein complex TonB ExbB–ExbD also existed. Due to that iron periplasm-to-cytosol transport requires ABC transporters on the cytosolic membrane, the transporter ATP-binding proteins are also upregulated to better contribute to the entry of iron into the cytoplasm as well as iron utilization for bacteria. Certainly, there are many complex regulatory mechanisms that were related to the use of iron, and we believe that our study will provide a good start for the iron regulation system in *A. paragallinarum*.

Furthermore, numerous cellular functions and signaling pathways were triggered or suppressed in *A. paragallinarum* under iron restriction in order to ensure iron homeostasis. Interestingly, several novel sRNAs were also elucidated in our study, while their expressions appeared to be different in *A. paragallinarum* under the iron-restriction condition compared with the iron-sufficiency condition. It suggests that these differences may reflect a survival strategy of *A. paragallinarum* in ensuring a rapid and accurate response to environmental iron changes. As far as we know, this is the first study of reporting comprehensive transcriptomic information in *A. paragallinarum* following iron restriction, with a bonus scene of the bioinformatic identification of new important sRNAs. In recent years, the iron-regulated proteins, especially the ferric siderophore outer membrane receptors, have been investigated as potential vaccine candidates (Alteri et al., 2009; Brumbaugh et al., 2013; Wang et al., 2019). Meaningfully, our study will also provide novel insight into the development of new antigens for potential vaccines against infectious coryza. The upregulated genes in *A. paragallinarum* under iron-restriction condition could be used for the preparation of gene-deleted bacterial strain and, thus, could be candidates for live attenuated vaccine and the genes themselves can also be regarded as candidates for subunit vaccines to control *A. paragallinarum* in poultry farms, just like other bacteria that have been previously reported (Zeng et al., 2009).

### Hut Protein Is Required for Iron Acquisition and Heme Utilization in *A. paragallinarum*

It is well known that heme utilization systems have essential roles in bacterial iron acquisition and pathogenicity (Shi et al., 2019). A heme uptake system generally consists of the TonB-dependent outer membrane receptor protein, periplasmic binding protein, and inner membrane-associated ABC transporter (Rio et al., 2005). Despite extensive studies on the mechanism of heme transport from outside to the cytoplasm, proteins that participated in heme utilization remain unknown. Nowadays, Hut (heme utilization) protein has been identified in *Vibrio cholerae* according to genome sequence and bioinformatics-based predictions (Uchida et al., 2019). Uchida et al. have recently purified and characterized HutZ as a heme-degrading enzyme in *V. cholerae*, which releases iron into the cytoplasm (Uchida et al., 2012). As a unique heme storage protein, HutZ is critical for heme utilization and can cleave heme to biliverdin *via* verdoheme (Uchida et al., 2019). Besides, it is reported that HutZ is also required for biofilm formation and contributes to the pathogenicity of *Edwardsiella piscicida* (Shi et al., 2019). HutX, a cytoplasmic heme transport protein for HutZ, facilitates the transfer of heme to HutZ *via* a specific protein–protein interaction (Sekine et al., 2016). In *A. paragallinarum*, the function of the Hut protein family has not yet been elucidated. In this study, HutZ and HutX proteins were also identified in *A. paragallinarum*. RNA-seq analysis and qPCR successfully confirmed the preferential expressions of these proteins in *A. paragallinarum* under iron-restriction condition, indicating that heme uptake may be an important route for iron acquisition in *A. paragallinarum*. Herein, our results provide a clue that the heme transport system can play an essential role in iron acquisition as well as HutZ and HutX proteins are considered to be necessary for heme utilization in *A. paragallinarum*. Just like other bacteria mentioned above, this HutZ in *A. paragallinarum* can also be considered as a heme-degrading enzyme that releases iron into the cytoplasm and HutX is regarded as a cytoplasmic heme transport protein for HutZ, which facilitates the iron acquisition and heme utilization in *A. paragallinarum*. These heme acquisition-associated genes might be potential candidate genes for the development of subunit vaccines to control *A. paragallinarum*. Nevertheless, the regulatory mechanism of heme utilization by HutZ and HutX is worthy of further investigation.

### Dominant Role of DUF Domain-Containing Proteins in *A. paragallinarum* Under Iron Restriction

Even when sequencing data is being increased, there are also a lot of genes or proteins that are uncharacterized and their domain functions are unknown (Nabi et al., 2020). These proteins are regarded as proteins having DUFs (Finn et al., 2016; El-Gebali et al., 2019). DUFs exist in a variety of species, such as around 1,500 in eukaryotes and 2,704 in bacteria (Goodacre et al., 2013). Evolutionary conservation suggests that many of the DUFs have vital functions. For example, DUF59, one of the most common DUFs, occurs in both bacteria and eukaryotes. Though the precise biochemical function of DUF59 remains unclear, previous studies have provided insights into its physiological roles. For instance, proteins containing DUF59 participate in many processes, which include maturation of iron–sulfur (FeS) proteins and intracellular iron homeostasis in *S. aureus* (Mashruwala et al., 2016). Recently, DUF143 has been considered to be essential in *E. coli* when cells are starved and its deletion in bacteria shows no obvious phenotype (Baba et al., 2006; Hauser et al., 2012). Until now, few studies characterize the DUF domain-containing proteins in *A. paragallinarum*. In this study, most of the genes encoding for numerous DUF domain-containing proteins were identified in *A. paragallinarum*. Among these proteins, majority of them were upregulated and only a few were downregulated in bacteria under the condition of iron restriction. Remarkably, DUF454, DUF4198, and DUF2318 domain-containing proteins were significantly promoted, while DUF1345 domain-containing protein was significantly suppressed in the iron-restriction group than in the control group. According to the sRNA analysis, we speculate that the expression pattern of several DUF domain-containing proteins could be regulated by sRNAs. This is the first research to characterize DUF domain-containing proteins in *A. paragallinarum*, implying that various DUF domain-containing proteins may have positive roles in transcriptional regulation of physiological processes to cope with environmental iron restriction. Further experimentation will be needed to scrutinize the molecular and functional aspects of these crucial DUF domain-containing proteins in detail.

### Stable Expression of Fic Protein Might Be Attributed to the Regulatory of sRNAs in *A. paragallinarum* Under Iron Restriction

Fic (filamentation induced by cAMP) protein is extensively present in bacteria, with a single member found in animals (Lu et al., 2016). It plays essential roles in drug tolerance, bacterial pathogenicity, and stress response. Previous studies have shown that Fic domain containing protein is able to post-translationally modify proteins (Mukherjee et al., 2011; Feng et al., 2012; Castro-Roa et al., 2013; Harms et al., 2016, 2017). In addition, Fic protein in non-pathogenic bacteria has been implicated in the regulation of bacterial cell division and programmed death (Worby et al., 2009). For instance, a mutation of the fic gene in *E. coli* can result in filamentation (Utsumi et al., 1982, 1983). Although Fic protein has attracted considerable interest for its functions in bacterial infections and stress responses, their functions and related molecular mechanisms in iron homeostasis in *A. paragallinarum* had been unknown until recently. Here, we found that this protein was present in *A. paragallinarum*. However, there was no significance of gene expression of this protein between the iron-restriction group and the control group. Thus, it seems that iron- restriction may not result in the abnormal expression of Fic protein in *A. paragallinarum*. Identification and bioinformatic characterization of sRNAs further confirmed that Fic protein could be regulated not only by upregulated sRNA0022 but also by downregulated sRNA0004 and sRNA0036 in bacteria under the iron-restriction condition compared with that under the control condition. Consequently, we speculate that Fic protein is still stably expressed in *A. paragallinarum* under iron restriction, which might be largely attributed to the existence of these regulatory sRNAs. Herein, our results demonstrate for the first time about the effects of iron restriction on the expression of Fic protein as well as the tight relationship between this protein and regulatory sRNAs in *A. paragallinarum*.

## Conclusion

We investigate the more profound transcriptomic profiles for the first time and illustrate changes of transcriptomes in *A. paragallinarum* in response to starvation using RNA-seq. Compared with iron sufficiency, iron restriction results in many transcriptomic changes including changes of global gene expression and induction of specific genes. The bioinformatic analyses demonstrate that the Hut protein and DUF domain-containing proteins are preferentially activated in *A. paragallinarum* following iron restriction and have a positive role in iron acquisition and heme utilization in response to iron starvation. In addition, several novel sRNAs are identified in *A. paragallinarum* and have potential regulatory roles in iron homeostasis, which is likely to facilitate the stable expression of Fic protein. Researches on these genes will enrich our knowledge about the mechanisms of growth and pathogenesis of *A. paragallinarum*, which can be regarded as potential targets for the development of new prophylactic and therapeutic measures against *A. paragallinarum*.

## Data Availability Statement

The data presented in the study are deposited in the (NCBI SRA) repository, accession number (SRR13291129).

## Author Contributions

CH, FX, FL, DL, ZH, and HS carried out the experiments, analyzed the data, and wrote the manuscript. CH, JL, and HS designed the study and supervised the project. CH, XZ, FX, GL, YH, JL, and HS assisted in the data analysis and discussion. CH and HS drew the figures. All authors read and approved the final manuscript.

## Conflict of Interest

The authors declare that the research was conducted in the absence of any commercial or financial relationships that could be construed as a potential conflict of interest.

## Footnotes

1 http://david.abcc.ncifcrf.gov/

2 http://cs.wellesley.edu/~btjaden/Rockhopper/

## References

[B2] Abascal E. N. Guerra A. C. Vazquez A. S. Tenorio V. R. Cruz C. V. Zenteno E. (2009). Identification of iron-acquisition proteins of *Avibacterium paragallinarum*. *Avian Pathol.* 38 209–213. 10.1080/03079450902912143 19468937

[B3] Alteri C. J. Hagan E. C. Sivick K. E. Smith S. N. Mobley H. L. T. (2009). Mucosal immunization with iron receptor antigens protects against urinary tract infection. *PLoS Pathogens* 5:e1000586. 10.1371/journal.ppat.1000586 19806177 PMC2736566

[B4] Alvarez-Estrada A. Gutierrez-Martin C. B. Rodriguez-Ferri E. F. Martinez-Martinez S. (2018). Transcriptomics of *Haemophilus (Glasserella) parasuis* serovar 5 subjected to culture conditions partially mimetic to natural infection for the search of new vaccine antigens. *BMC Vet. Res.* 14:326. 10.1186/s12917-018-1647-1641 PMC6219065 30400794

[B5] Anderson M. T. Armstrong S. K. (2004). The BfeR regulator mediates enterobactin-inducible expression of *Bordetella enterobactin* utilization genes. *J. Bacteriol.* 186 7302–7311. 10.1128/JB.186.21.7302-7311.2004 15489442 PMC523226

[B6] Baba T. Ara T. Hasegawa M. Takai Y. Okumura Y. Baba M. (2006). Construction of *Escherichia coli* K-12 in-frame, single-gene knockout mutants: the Keio collection. *Mol. Syst. Biol.* 2:2006.0008. 10.1038/msb4100050 16738554 PMC1681482

[B7] Barnard T. G. Van Heerden E. Bragg R. R. Albertyn J. (2008). Haemophilus paragallinarum haemagglutinin: role in adhesion, serotyping and pathogenicity. *Onderstepoort J. Vet. Res.* 75 11–16. 18575059

[B8] Blackall P. J. (1999). Infectious coryza: overview of the disease and new diagnostic options. *Clin. Microbiol. Rev.* 12 627–632. 10.1128/cmr.12.4.627 10515906 PMC88928

[B9] Boucher C. E. Theron C. W. Jansen A. C. Bragg R. R. (2014). Transcriptional profiling of chicken immunity-related genes during infection with *Avibacterium paragallinarum*. *Vet. Immunol. Immunopathol.* 158 135–142. 10.1016/j.vetimm.2013.12.004 24613002

[B10] Bragg R. R. (2005). Effects of differences in virulence of different serovars of *Haemophilus paragallinarum* on perceived vaccine efficacy. *Onderstepoort J. Vet. Res.* 72 1–6. 10.4102/ojvr.v72i1.218 15991700

[B11] Bragg R. R. Coetzee L. Verschoor J. A. (1996). Changes in the incidences of the different serovars of *Haemophilus paragallinarum* in South Africa: a possible explanation for vaccination failures. *Onderstepoort J. Vet. Res.* 63 217–226. 8917859

[B12] Braun V. Hantke K. Koster W. (1998). Bacterial iron transport: mechanisms, genetics, and regulation. *Met. Ions Biol. Syst.* 35 67–145. 9444760

[B13] Brumbaugh A. R. Smith S. N. Mobley H. L. T. (2013). Immunization with the Yersiniabactin receptor. FyuA, protects against pyelonephritis in a murine model of urinary tract infection. *Infect. Immun.* 81 3309–3316. 10.1128/Iai.00470-413 23798537 PMC3754202

[B14] Castro-Roa D. Garcia-Pino A. De Gieter S. van Nuland N. A. J. Loris R. Zenkin N. (2013). The fic protein doc uses an inverted substrate to phosphorylate and inactivate EF-Tu. *Nat. Chem. Biol.* 9 811–817. 10.1038/nchembio.1364 24141193 PMC3836179

[B15] Chareyre S. Mandin P. (2018). Bacterial iron homeostasis regulation by sRNAs. *Microbiol. Spectr.* 6 1–15. 10.1128/microbiolspec.RWR-0010-2017 29573257 PMC11633579

[B16] Chu B. C. Garcia-Herrero A. Johanson T. H. Krewulak K. D. Lau C. K. Peacock R. S. (2010). Siderophore uptake in bacteria and the battle for iron with the host; a bird’s eye view. *Biometals* 23 601–611. 10.1007/s10534-010-9361-x 20596754

[B17] Contreras H. Chim N. Credali A. Goulding C. W. (2014). Heme uptake in bacterial pathogens. *Curr. Opin. Chem. Biol.* 19 34–41. 10.1016/j.cbpa.2013.12.014 24780277 PMC4007353

[B18] del Rio M. L. Gutierrez-Martin C. B. Rodriguez-Barbosa J. I. Navas J. Rodriguez-Ferri E. F. (2005). Identification and characterization of the TonB region and its role in transferrin-mediated iron acquisition in *Haemophilus parasuis*. *FEMS Immunol. Med. Microbiol.* 45 75–86. 10.1016/j.femsim.2005.02.008 15985226

[B19] El Mouali Y. Gaviria-Cantin T. Sanchez-Romero M. A. Gibert M. Westermann A. J. Vogel J. (2018). CRP-cAMP mediates silencing of *Salmonella virulence* at the post-transcriptional level. *PLoS Genet.* 14:e1007401. 10.1371/journal.pgen.1007401 29879120 PMC5991649

[B20] El-Gebali S. Mistry J. Bateman A. Eddy S. R. Luciani A. Potter S. C. (2019). The Pfam protein families database in 2019. *Nucleic Acids Res.* 47 D427–D432. 10.1093/nar/gky995 30357350 PMC6324024

[B21] Feng F. Yang F. Rong W. Wu X. Zhang J. Chen S. (2012). A *Xanthomonas uridine* 5’-monophosphate transferase inhibits plant immune kinases. *Nature* 485 114–118. 10.1038/nature10962 22504181

[B22] Finn R. D. Coggill P. Eberhardt R. Y. Eddy S. R. Mistry J. Mitchell A. L. (2016). The Pfam protein families database: towards a more sustainable future. *Nucleic Acids Res.* 44 D279–D285. 10.1093/nar/gkv1344 26673716 PMC4702930

[B23] Gerrick E. R. Barbier T. Chase M. R. Xu R. Francois J. Lin V. H. (2018). Small RNA profiling in *Mycobacterium tuberculosis* identifies MrsI as necessary for an anticipatory iron sparing response. *Proc. Natl. Acad. Sci. U S A.* 115 6464–6469. 10.1073/pnas.1718003115 29871950 PMC6016810

[B24] Goodacre N. F. Gerloff D. L. Uetz P. (2013). Protein domains of unknown function are essential in bacteria. *mBio* 5:e00744-13. 10.1128/mBio.00744-713 PMC3884060 24381303

[B25] Harms A. Liesch M. Korner J. Quebatte M. Engel P. Dehio C. (2017). A bacterial toxin-antitoxin module is the origin of inter-bacterial and inter-kingdom effectors of *Bartonella*. *PLoS Genet.* 13:e1007077. 10.1371/journal.pgen.1007077 29073136 PMC5675462

[B26] Harms A. Stanger F. V. Dehio C. (2016). Biological diversity and molecular plasticity of FIC domain proteins. *Annu. Rev. Microbiol.* 70 341–360. 10.1146/annurev-micro-102215-195245 27482742

[B27] Hauser R. Pech M. Kijek J. Yamamoto H. Titz B. Naeve F. (2012). RsfA (YbeB) proteins are conserved ribosomal silencing factors. *PLoS Genet.* 8:e1002815. 10.1371/journal.pgen.1002815 22829778 PMC3400551

[B28] Higgs P. I. Myers P. S. Postle K. (1998). Interactions in the TonB-dependent energy transduction complex: ExbB and ExbD form homomultimers. *J. Bacteriol.* 180 6031–6038. 10.1128/JB.180.22.6031-6038.1998 9811664 PMC107680

[B29] Horiyama T. Nishino K. (2014). AcrB, AcrD, and MdtABC multidrug efflux systems are involved in enterobactin export in *Escherichia coli*. *PLoS One* 9:e108642. 10.1371/journal.pone.0108642 25259870 PMC4178200

[B30] Huo C. Xiao J. Xiao K. Zou S. Wang M. Qi P. (2020). Pre-Treatment with zirconia nanoparticles reduces inflammation induced by the pathogenic H5N1 influenza virus. *Int. J. Nanomed.* 15 661–674. 10.2147/IJN.S221667 32099358 PMC6996547

[B31] Koeppen K. Hampton T. H. Jarek M. Scharfe M. Gerber S. A. Mielcarz D. W. (2016). A novel mechanism of host-pathogen interaction through sRNA in bacterial outer membrane vesicles. *PLoS Pathog* 12:e1005672. 10.1371/journal.ppat.1005672 27295279 PMC4905634

[B32] Larsen R. A. Thomas M. G. Wood G. E. Postle K. (1994). Partial suppression of an *Escherichia coli* TonB transmembrane domain mutation (delta V17) by a missense mutation in ExbB. *Mol. Microbiol.* 13 627–640. 10.1111/j.1365-2958.1994.tb00457.x 7997175

[B33] Lu C. Nakayasu E. S. Zhang L. Q. Luo Z. Q. (2016). Identification of Fic-1 as an enzyme that inhibits bacterial DNA replication by AMPylating GyrB, promoting filament formation. *Sci. Signal.* 9:ra11. 10.1126/scisignal.aad0446 26814232

[B34] Mashruwala A. A. Bhatt S. Poudel S. Boyd E. S. Boyd J. M. (2016). The DUF59 containing protein SufT is involved in the maturation of iron-sulfur (FeS) proteins during conditions of high FeS cofactor demand in *Staphylococcus aureus*. *PLoS Genet.* 12:e1006233. 10.1371/journal.pgen.1006233 27517714 PMC4982691

[B35] Masse E. Vanderpool C. K. Gottesman S. (2005). Effect of RyhB small RNA on global iron use in *Escherichia coli*. *J. Bacteriol.* 187 6962–6971. 10.1128/JB.187.20.6962-6971.2005 16199566 PMC1251601

[B36] Miksch G. Dobrowolski P. (1995). Growth phase-dependent induction of stationary-phase promoters of *Escherichia coli* in different gram-negative bacteria. *J. Bacteriol.* 177 5374–5378. 10.1128/jb.177.18.5374-5378.1995 7665531 PMC177338

[B37] Mukherjee S. Liu X. Arasaki K. McDonough J. Galan J. E. Roy C. R. (2011). Modulation of rab GTPase function by a protein phosphocholine transferase. *Nature* 477 103–106. 10.1038/nature10335 21822290 PMC3206611

[B38] Nabi R. B. S. Tayade R. Imran Q. M. Hussain A. Shahid M. Yun B. W. (2020). Functional insight of nitric-oxide induced DUF genes in *Arabidopsis thaliana*. *Front. Plant Sci.* 11:1041. 10.3389/fpls.2020.01041 32765550 PMC7378322

[B39] Palyada K. Threadgill D. Stintzi A. (2004). Iron acquisition and regulation in Campylobacter jejuni. *J. Bacteriol.* 186 4714–4729. 10.1128/Jb.186.14.4714-4729.2004 15231804 PMC438614

[B40] Parrow N. L. Fleming R. E. Minnick M. F. (2013). Sequestration and scavenging of iron in infection. *Infect. Immun.* 81 3503–3514. 10.1128/IAI.00602-613 23836822 PMC3811770

[B41] Postle K. (1993). TonB protein and energy transduction between membranes. *J. Bioenerg. Biomembr.* 25 591–601. 10.1007/BF00770246 8144488

[B42] Reinhart A. A. Powell D. A. Nguyen A. T. O’Neill M. Djapgne L. Wilks A. (2015). The prrF-encoded small regulatory RNAs are required for iron homeostasis and virulence of *Pseudomonas aeruginosa*. *Infect. Immun.* 83 863–875. 10.1128/IAI.02707-2714 25510881 PMC4333466

[B43] Repoila F. Darfeuille F. (2009). Small regulatory non-coding RNAs in bacteria: physiology and mechanistic aspects. *Biol. Cell* 101 117–131. 10.1042/BC20070137 19076068

[B44] Richard K. L. Kelley B. R. Johnson J. G. (2019). Heme uptake and utilization by gram-negative bacterial pathogens. *Front. Cell Infect. Microbiol.* 9:81. 10.3389/fcimb.2019.00081 30984629 PMC6449446

[B45] Rio S. J. Osorio C. R. Lemos M. L. (2005). Heme uptake genes in human and fish isolates of Photobacterium damselae: existence of hutA pseudogenes. *Arch. Microbiol.* 183 347–358. 10.1007/s00203-005-0779-774 15918073

[B46] Rouault T. A. (2004). Microbiology. pathogenic bacteria prefer heme. *Science* 305 1577–1578. 10.1126/science.1102975 15361615

[B47] Sekine Y. Tanzawa T. Tanaka Y. Ishimori K. Uchida T. (2016). Cytoplasmic heme-binding protein (HutX) from *Vibrio cholerae* is an intracellular heme transport protein for the heme-degrading enzyme, hutZ. *Biochemistry* 55 884–893. 10.1021/acs.biochem.5b01273 26807477

[B48] Shi Y. J. Fang Q. J. Huang H. Q. Gong C. G. Hu Y. H. (2019). HutZ is required for biofilm formation and contributes to the pathogenicity of *Edwardsiella piscicida*. *Vet. Res.* 50:76. 10.1186/s13567-019-0693-694 PMC6775658 31578154

[B49] Siegele D. A. Kolter R. (1992). Life after log. *J. Bacteriol.* 174 345–348. 10.1128/jb.174.2.345-348.1992 1729229 PMC205722

[B50] Skaar E. P. Humayun M. Bae T. DeBord K. L. Schneewind O. (2004). Iron-source preference of *Staphylococcus aureus* infections. *Science* 305 1626–1628. 10.1126/science.1099930 15361626

[B51] Sun H. Xie S. Li X. Xu F. Li Y. Boucher C. E. (2018). Selection of *Avibacterium paragallinarum* page serovar B strains for an infectious coryza vaccine. *Vet. Immunol. Immunopathol.* 199 77–80. 10.1016/j.vetimm.2018.04.001 29678233

[B52] Thulasiraman P. Newton S. M. Xu J. Raymond K. N. Mai C. Hall A. (1998). Selectivity of ferric enterobactin binding and cooperativity of transport in gram-negative bacteria. *J. Bacteriol.* 180 6689–6696. 10.1128/JB.180.24.6689-6696.1998 9852016 PMC107775

[B53] Uchida T. Dojun N. Ota K. Sekine Y. Nakamura Y. Umetsu S. (2019). Role of conserved arginine in the heme distal site of HutZ from *Vibrio cholerae* in the heme degradation reaction. *Arch. Biochem. Biophys.* 677:108165. 10.1016/j.abb.2019.108165 31689379

[B54] Uchida T. Sekine Y. Matsui T. Ikeda-Saito M. Ishimori K. (2012). A heme degradation enzyme, HutZ, from *Vibrio cholerae*. *Chem. Commun. (Camb)* 48 6741–6743. 10.1039/c2cc31147j 22627893

[B55] Utsumi R. Kawamukai M. Obata K. Morita J. Himeno M. Komano T. (1983). Identification of a membrane protein induced concurrently with cell filamentation by cyclic AMP in an *Escherichia coli* K-12 fic mutant. *J. Bacteriol.* 155 398–401. 10.1128/JB.155.1.398-401.1983 6305918 PMC217692

[B56] Utsumi R. Nakamoto Y. Kawamukai M. Himeno M. Komano T. (1982). Involvement of cyclic AMP and its receptor protein in filamentation of an *Escherichia coli* fic mutant. *J. Bacteriol.* 151 807–812. 10.1128/JB.151.2.807-812.1982 6284712 PMC220329

[B57] Wang Y. Q. Wang X. Y. Ali F. Li Z. Q. Fu Y. Y. Yang X. J. (2019). Comparative extracellular proteomics of *Aeromonas hydrophila* reveals iron-regulated secreted proteins as potential vaccine candidates. *Front. Immunol.* 10:256. 10.3389/fimmu.2019.00256 30833947 PMC6387970

[B58] Wen S. Chen X. Xu F. Sun H. (2016). Validation of reference genes for real-time quantitative PCR (qPCR) analysis of *Avibacterium paragallinarum*. *PLoS One* 11:e0167736. 10.1371/journal.pone.0167736 27942007 PMC5152862

[B59] Williams P. Morton D. J. Towner K. J. Stevenson P. Griffiths E. (1990). Utilization of enterobactin and other exogenous iron sources by *Haemophilus influenzae*, H. *parainfluenzae* and *H. paraphrophilus*. *J. Gen. Microbiol.* 136 2343–2350. 10.1099/00221287-136-12-2343 2150414

[B60] Worby C. A. Mattoo S. Kruger R. P. Corbeil L. B. Koller A. Mendez J. C. (2009). The fic domain: regulation of cell signaling by adenylylation. *Mol. Cell* 34 93–103. 10.1016/j.molcel.2009.03.008 19362538 PMC2820730

[B61] Xu F. Zeng X. Haigh R. D. Ketley J. M. Lin J. (2010). Identification and characterization of a new ferric enterobactin receptor, CfrB, in Campylobacter. *J. Bacteriol.* 192 4425–4435. 10.1128/JB.00478-410 20585060 PMC2937379

[B62] Xu Y. Cheng J. Huang X. Xu M. Feng J. Liu C. (2019). Characterization of emergent *Avibacterium paragallinarum* strains and the protection conferred by infectious coryza vaccines against them in China. *Poult. Sci.* 98 6463–6471. 10.3382/ps/pez531 31801310 PMC8913951

[B63] Ying B. W. Matsumoto Y. Kitahara K. Suzuki S. Ono N. Furusawa C. (2015). Bacterial transcriptome reorganization in thermal adaptive evolution. *BMC Genom.* 16:802. 10.1186/s12864-015-1999-x 26474851 PMC4609109

[B64] Zeng X. Xu F. Lin J. (2009). Molecular, antigenic, and functional characteristics of ferric enterobactin receptor CfrA in *Campylobacter jejuni*. *Infect. Immun.* 77 5437–5448. 10.1128/IAI.00666-669 19737895 PMC2786435

[B65] Zhang P. J. Miao M. Sun H. Gong Y. Blackall P. J. (2003). Infectious coryza due to *Haemophilus paragallinarum* serovar B in China. *Aust. Vet. J.* 81 96–97. 10.1111/j.1751-0813.2003.tb11445.x 15084021

[B66] Zhu H. Xie G. Liu M. Olson J. S. Fabian M. Dooley D. M. (2008). Pathway for heme uptake from human methemoglobin by the iron-regulated surface determinants system of *Staphylococcus aureus*. *J. Biol. Chem.* 283 18450–18460. 10.1074/jbc.M801466200 18467329 PMC2440603

