# Supplementary material for: The Transcriptomic and Bioinformatic Characterizations of Iron Acquisition and Heme Utilization in Avibacterium paragallinarum in Response to Iron-Starvation
Source: Front Microbiol. 2021 Mar 4;12:610196. doi: 10.3389/fmicb.2021.610196 (PMC7970244; doi:10.3389/fmicb.2021.610196)
Supplement: Supplementary file 1 [file Data_Sheet_1.docx]

**Supporting information**

**The transcriptomic and bioinformatic characterizations of iron acquisition and haem utilization in *Avibacterium paragallinarum* in response to iron-starvation**

Caiyun Huo^1^, Ximin Zeng^2^, Fuzhou Xu^1^, Fangbing Li^1^, Donghai Li^1,3^, Guiping Li^1^, Zhenguo Hu^1^, Yanxin Hu^3^, Jun Lin^2^, Huiling Sun^1*^

^1^ Beijing Key Laboratory for Prevention and Control of Infectious Diseases in Livestock and Poultry, Institute of Animal Husbandry and Veterinary Medicine, Beijing Academy of Agriculture and Forestry Sciences, Beijing, China.

^2^ Department of Animal Science, The University of Tennessee, 2505 River Drive, Knoxville, Tennessee, America.

^3^ Key Laboratory of Animal Epidemiology of Ministry of Agriculture, College of Veterinary Medicine, China Agricultural University, Beijing, China.

***** **Corresponding author:**

Dr. Huiling Sun, Beijing Key Laboratory for Prevention and Control of Infectious Diseases in Livestock and Poultry, Institute of Animal Husbandry and Veterinary Medicine, Beijing Academy of Agriculture and Forestry Science, #9 Shuguang Huayuan Zhonglu, Haidian District, Beijing, 100097, China. Email: sunhuiling01@163.com

**Table S1. The primer sequences**

| Target name | Primers |
| --- | --- |
| VY92_RS06600 | 5’- GGGCAGCACAGTTTTTATCC -3’ |
|  | 5’- GTCAGCTCAATGACTTGTTT -3’ |
| VY92_RS09125 | 5’- GTAAATTCCACGGTACGCAT -3’ |
|  | 5’- TTTGTCCGAGAAAATATTGC -3’ |
| VY92_RS09790 | 5’- CGTGATTTAGTGGTGGATCT -3’ |
|  | 5’- TAGCTGTGCTGGGGTTTGTT -3’ |
| VY92_RS01655 | 5’- AACTTCGCACCCATCATTTC -3’ |
|  | 5’- CCCCAAAACCACTATATCTT -3’ |
| VY92_RS03730 | 5’- ATGGCAAGGTGTCTTTAATG -3’ |
|  | 5’- CGTTGTTGCAGTAAGCCTGT -3’ |
| VY92_RS03735 | 5’- GGGTTCGATCTTTGAAGTAT -3’ |
|  | 5’- CTGATAAGGGCAATGTCTTT -3’ |
| VY92_RS00335 | 5’- CTTTTTGGCGATTGAAACAG -3’ |
|  | 5’- ACATCAACAAAATCACTGCG -3’ |
| VY92_RS05725 | 5’- TCTCAAGGCTCTGCCCTTTA -3’ |
|  | 5’- GCCTGAATCTCACGAATGGT -3’ |
| gyrA | 5’- AGTGAGCGTAACGGCAAAGT -3’ |
|  | 5’- ATGTCCGATTCTTCGTCGTC -3’ |

**Table S2. The OD values of cultures**

|  | 0h | 3.5h | 5h | 6h |
| --- | --- | --- | --- | --- |
| C | 0.0400 | 0.2967 | 0.8605 | 1.2319 |
| R | 0.0427 | 0.2750 | 0.7233 | 1.1210 |

Note: C represents the control group, R represents the iron-restriction group.

**Fig. S1: Experimental design schematic for** **RNA-seq.** The strain 3005 of the *Av. paragallinarum* serovar C was used in this study. TSB and TSA that were added with 10% chicken serum and 0.0025% NAD were used for propagation and maintenance of the strain. Then, *Av. paragallinarum* was grown under in vitro culture conditions with iron-restriction (R) or without iron-restriction (C, as a control). For iron-restriction condition, *Av. paragallinarum* cells were grown in 200ml MEMα with 0.0025% (w/v) NAD. For control condition, extra 40μM FeSO4 was added in the cultures. The cultures were grown in a shaking incubator at 37˚C until reaching an OD of 0.6 at 600 nm, and samples were harvested by centrifugation, followed immediately resuspended in RNAlater. Then, RNA was isolated for RNA-seq.

**
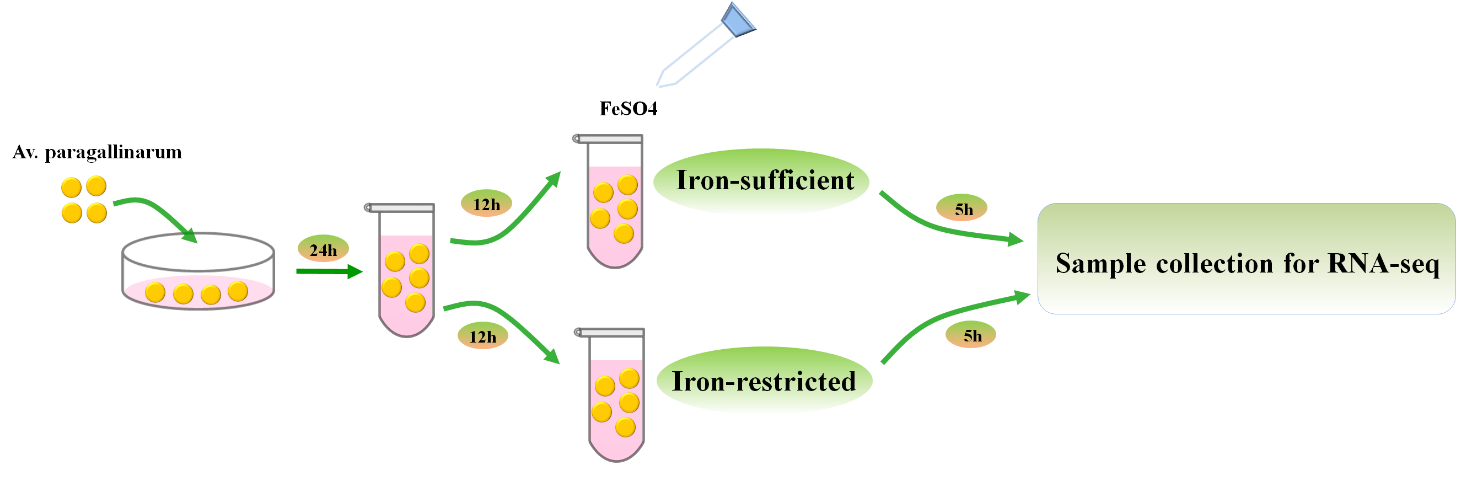
**

**Fig. S2: The nucleotide distribution of reads in *Av. paragallinarum* under conditions with iron-restriction or without iron-restriction.** (A and B) The nucleotide distribution of raw and clean data was analyzed, respectively. R_1, R_2 and R_3 represent the three independent replicates in iron-restriction group. C_1, C_2 and C_3 represent the three independent replicates in control group.


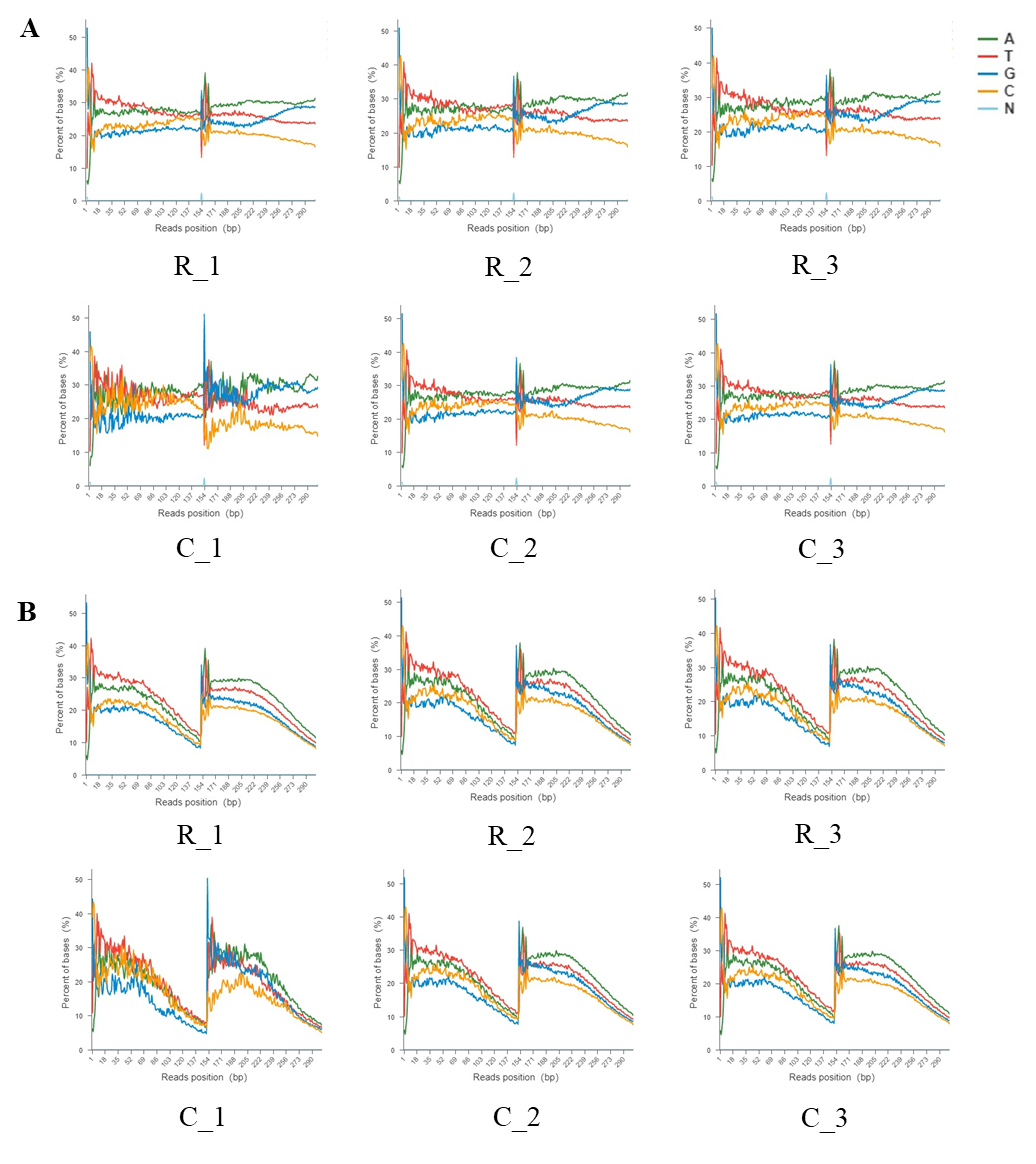


**Fig. S3: The** **screened DEGs in *Av. paragallinarum* under conditions with iron-restriction and without iron-restriction.** The heat map presenting the screened DEGs in *Av. paragallinarum* under control condition compared to iron-restriction condition (control vs iron-restriction). R_1, R_2 and R_3 represent the three independent replicates in iron-restriction group. C_1, C_2 and C_3 represent the three independent replicates in control group.


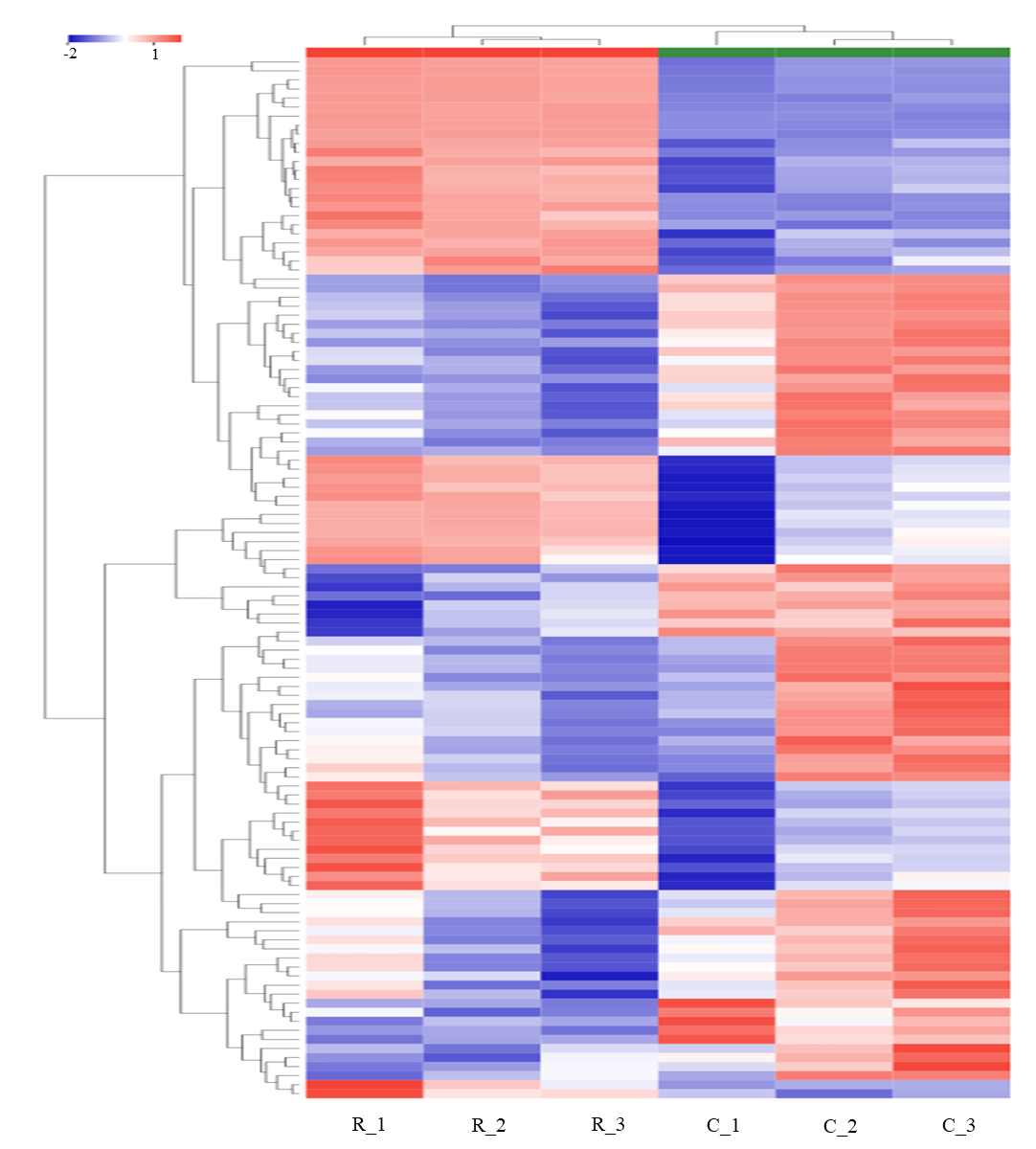


**Fig. S4: Validation of VY92_RS05725 in *Av. paragallinarum* under iron-restriction condition compared to control condition by qPCR.** The mRNA levels of VY92_RS05725 in *Av. paragallinarum* under iron-restriction condition compared to control condition were determined by real-time PCR (N=3), respectively. ns=no significance.


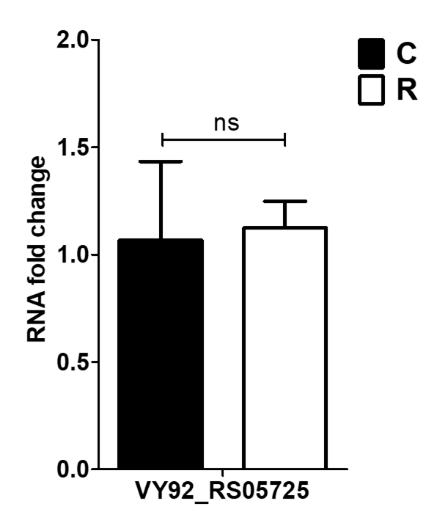


**Data Set S1: Summary of transcripts in *Av. paragallinarum* under conditions with iron-restriction and without iron-restriction.**

**Data Set S2: Summary of** **GO annotations analysis of DEGs in *Av. paragallinarum* under iron-restriction condition compared to control condition.**

**Data Set S3: Summary of** **GO enrichment analysis of up-regulated genes in *Av. paragallinarum* under iron-restriction condition compared to control condition.**

**Data Set S4: Summary of** **GO enrichment analysis of down-regulated genes in *Av. paragallinarum* under iron-restriction condition compared to control condition.**

**Data Set S5: Summary of** **KEGG enrichment analysis of up-regulated genes in *Av. paragallinarum* under iron-restriction condition compared to control condition.**

**Data Set S6: Summary of** **KEGG enrichment analysis of down-regulated genes in *Av. paragallinarum* under iron-restriction condition compared to control condition.**

**Data Set S7: Summary of** **the length of all sRNAs in *Av. paragallinarum*.**

**Data Set S8: Summary of** **the genes targeted by several regulatory sRNAs in *Av. paragallinarum* under iron-restriction condition** **compared to control condition.**
